# Supplementary figures and images for: Alpha-, beta-, and gamma-diversity of bacteria varies across habitats
Source: PLoS One. 2020 Sep 23;15(9):e0233872. doi: 10.1371/journal.pone.0233872 (PMC7510982; doi:10.1371/journal.pone.0233872)

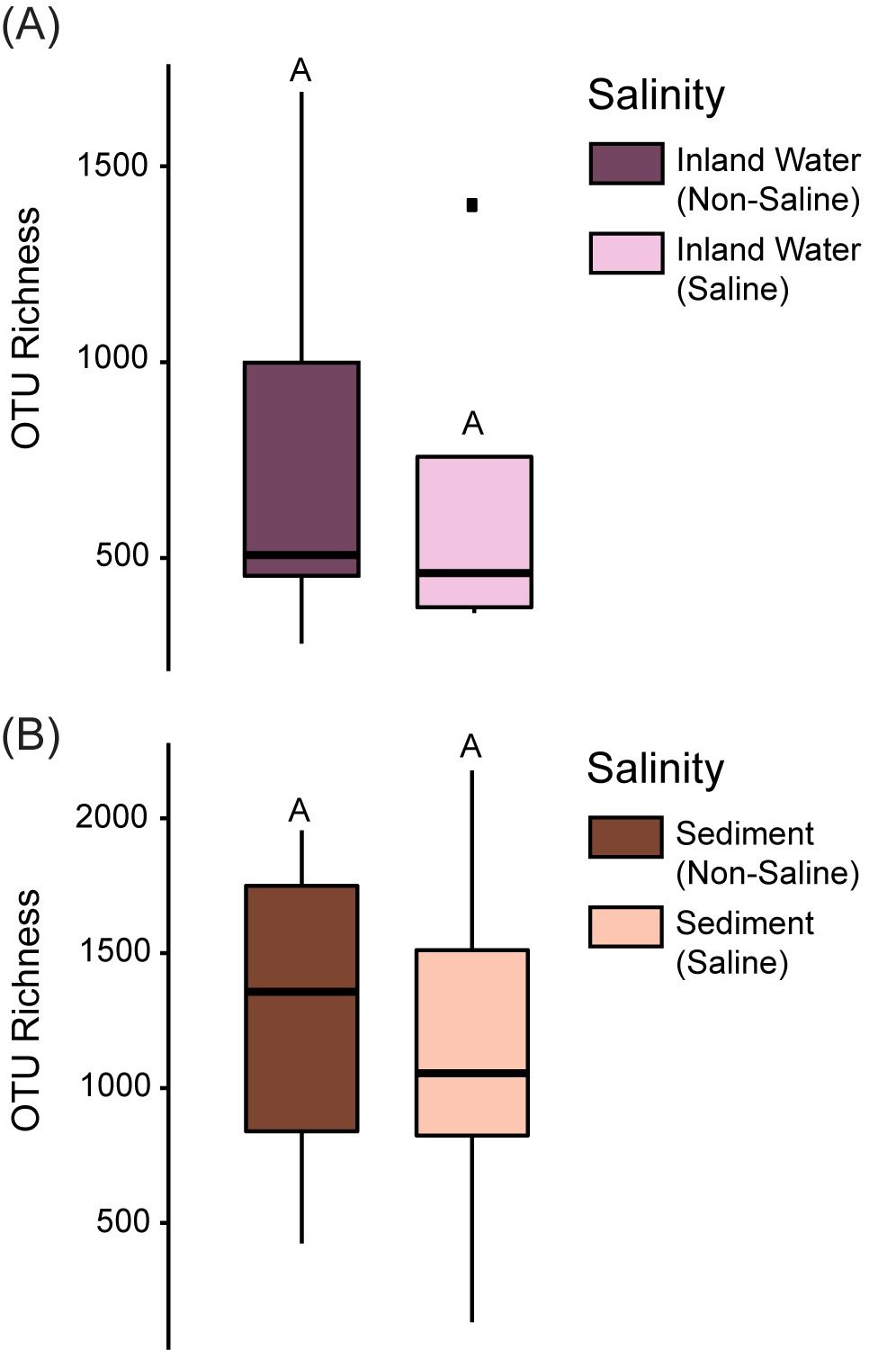

Supplement: S1 Fig — Taxon richness did not differ between saline and non-saline samples from inland water or sediment habitats. (TIF) [file pone.0233872.s001.tif]

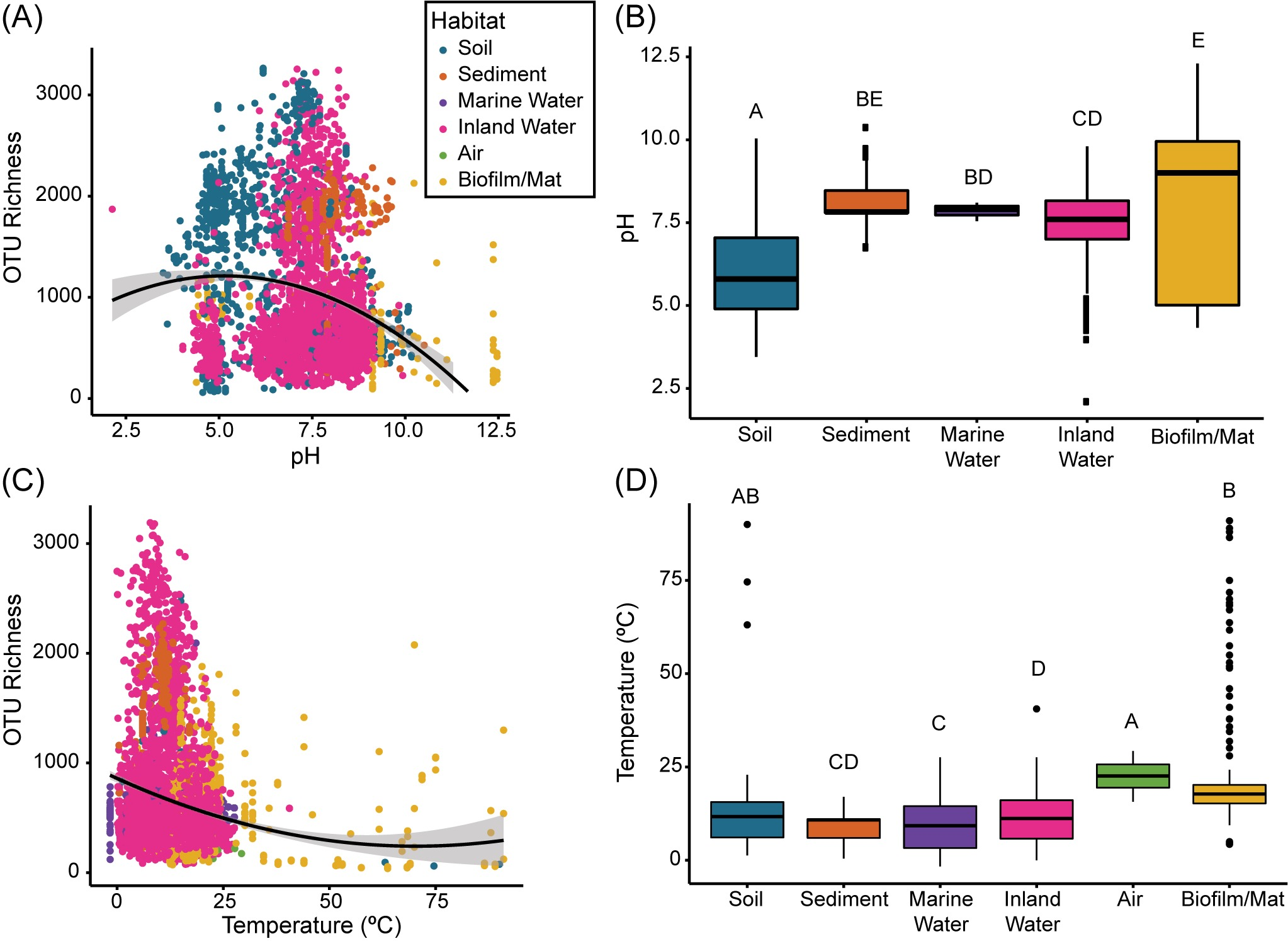

Supplement: S2 Fig — (A) pH significantly impacts taxon richness (P < 0.0001). Each point represents an individual EMP sample (not a geocluster) and is colored by habitat. (B) pH differs among habitats (ANOVA, p < 0.0001). Out of all the EMP environmental samples used in this study, 30.7% had associated pH metadata: 0% of air samples, 60.0% of inland water samples, 19.6% of sediment samples, 6.5% of marine water samples, 5.1% of biofilm/mat samples, and 23.4% of soil samples. (C) Temperature significantly influences taxon richness (P < 0.0001). Each point represents an individual EMP sample (not a geocluster) and is colored by habitat. (D) Temperature recorded when samples were collected (EMP metadata) differs among habitats (ANOVA, p < 0.0001). Out of all the EMP environmental samples used in this study, 47.3% had associated temperature metadata: 9.0% of air samples, 77.0% of inland water samples, 17.5% of sediment samples, 48.8% of marine water samples, 81.0% of biofilm/mat samples, and 0.6% of soil samples. (TIF) [file pone.0233872.s002.tif]

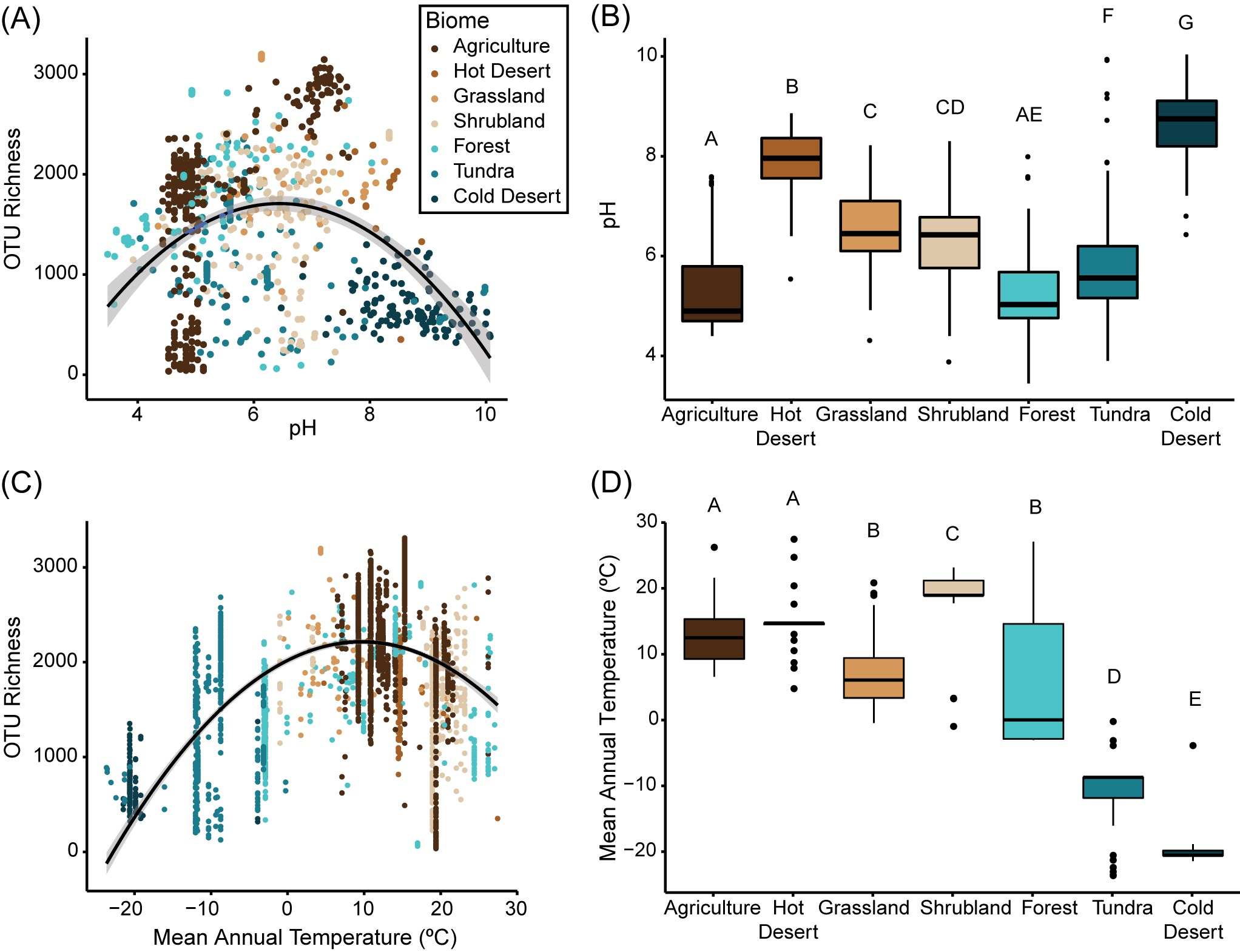

Supplement: S3 Fig — (A) pH significantly impacts taxon richness (P = 0.001). Each point represents an individual EMP soil sample (not a geocluster) and is colored by biome. (B) pH differs among biomes (ANOVA, p < 0.0001). (C) Mean annual temperature significantly impacts taxon richness (P < 0.0001). Each point represents an individual EMP soil sample (not a geocluster) and is colored by biome. Temperature data was retrieved from WorldClim, a publicly available data source. (D) Temperature differs among biomes (ANOVA, p < 0.0001). (TIF) [file pone.0233872.s003.tif]

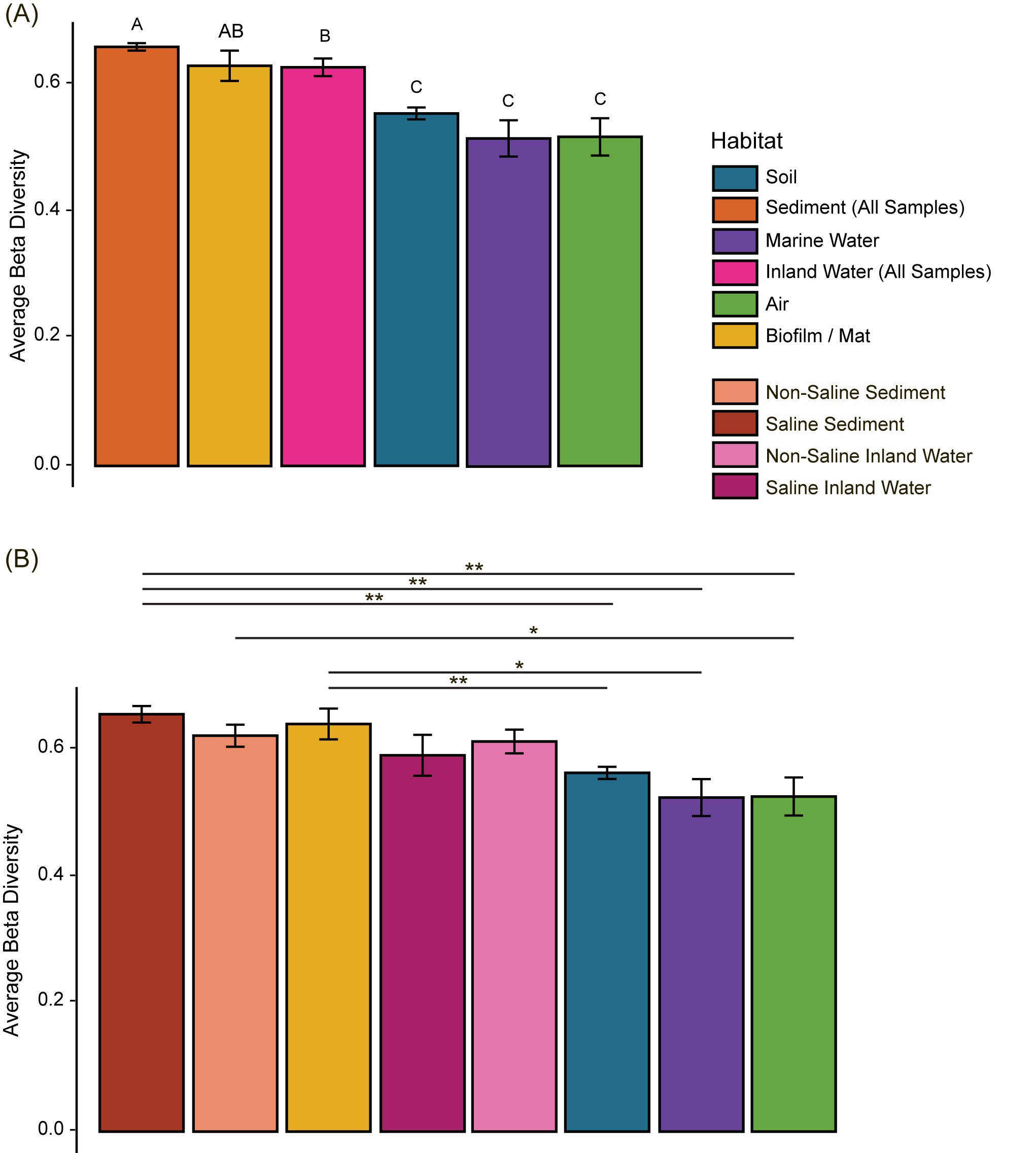

Supplement: S4 Fig — The level of beta-diversity within sediments and inland water is not driven by the combination of saline and non-saline samples within a single habitat. (A) Mean beta-diversity (distance from centroid) ± standard error of the six habitats, ranking habitats from highest to lowest beta-diversity. (B) Mean beta-diversity ± standard error of the six habitats with sediment and inland water habitats split into saline and non-saline samples. (TIF) [file pone.0233872.s004.tif]

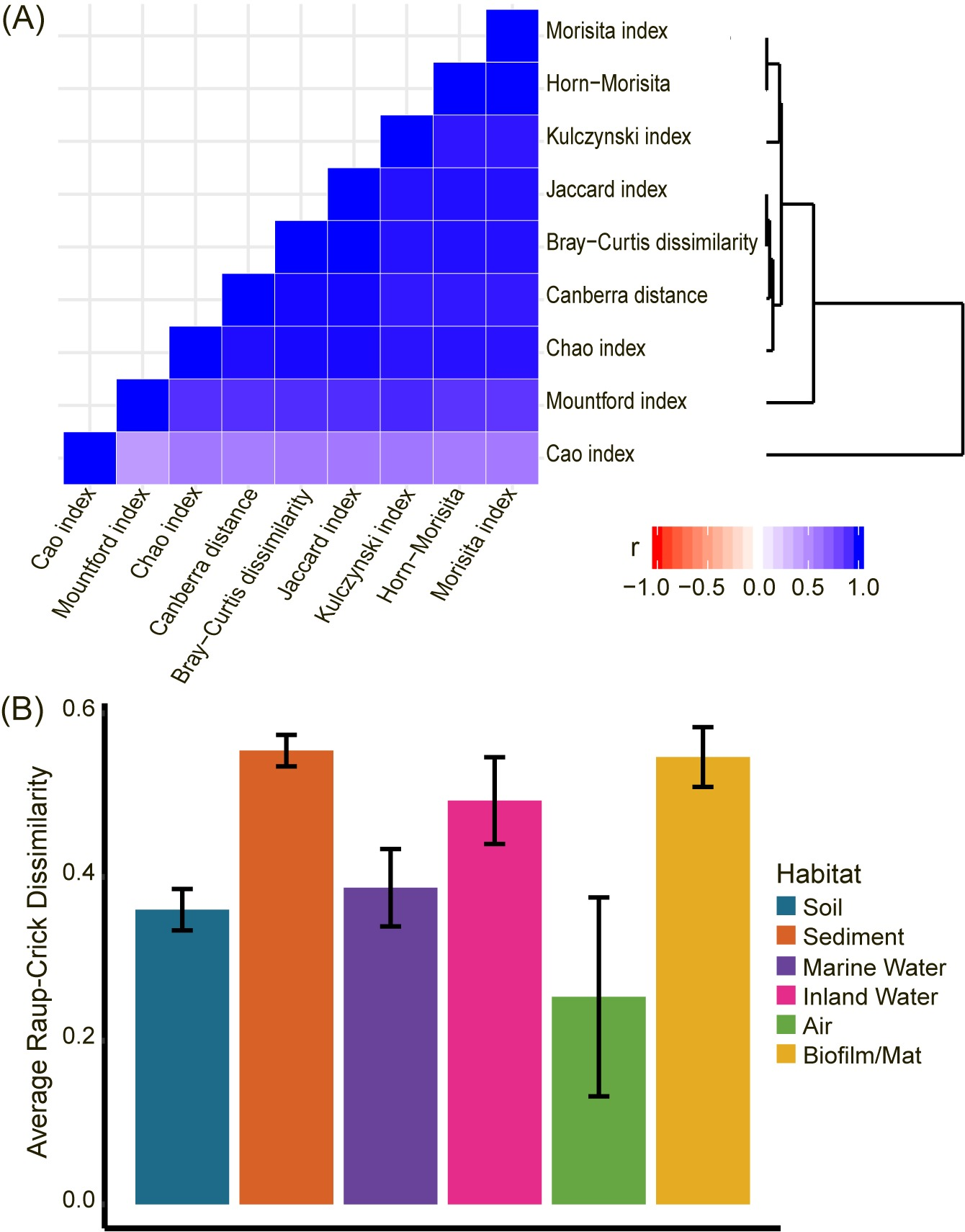

Supplement: S5 Fig — Beta-diversity patterns are not dependent on the beta-diversity metric used. (A) Heatmap shows degree of correlation (r from a Spearman’s mantel test with all EMP samples used in analysis). Dendrogram shows relatedness of metrics based on their correlation strength. (B) Mean Raup-Crick dissimilarity (distance from centroid) ± standard error. The patterns shown by Raup-Crick analysis match those demonstrated by Bray-Curtis metric. (TIF) [file pone.0233872.s005.tif]
