# Supplementary material for: Alpha-, beta-, and gamma-diversity of bacteria varies across habitats
Source: PLoS One. 2020 Sep 23;15(9):e0233872. doi: 10.1371/journal.pone.0233872 (PMC7510982; doi:10.1371/journal.pone.0233872)
Supplement: S1 Appendix — (DOCX) [file pone.0233872.s006.docx]

## S1 Appendix. Sample Locations

Sediment samples came from freshwater ponds/lakes and rivers/streams, saline ponds/lakes, coastal, mangrove, and oil-contaminated marine sediments. Marine samples came from northern seas to ocean waters near the equator. Inland water samples included rivers/streams, non-saline lakes/ponds, saline lakes/ponds, well water, groundwater, and sinkholes. Air samples were taken from urban regions in North America and Europe. Biofilm/mat samples were from kelp forests, hot springs, volcanic geothermal vents, coral reefs, Arctic lakes/streams, marine and travertine hydrothermal vents, tundra peat soils, and intertidal regions.

Agricultural soils included samples from vineyards, fertilized temperate grasslands, soybean fields, wheat fields, rice paddies, a legume cropland, a palm plantation, green roofs, an urban park, a coffee plantation, a FACE pasture, a potato farm, a maize field, and a fertilized urban field soil. Grassland soils are from North America, Hawai’i, and Argentina. Shrubland soils came from desert shrublands, mountain shrublands, and tropical volcanic shrublands. Forest soils are from taiga, temperature forests, tropical forests, volcanic forests from Hawaii, and unknown forests. Hot desert soils are from North America and India. Cold desert soils are from Antarctica. Tundra soils are from North America, Asia, Europe, Greenland, and Antarctica.
